# Sleep disorders in a sample of Lebanese children: the role of parental mental health and child nutrition and activity

**DOI:** 10.1186/s12887-021-02795-w

**Published:** 2021-07-23

**Authors:** Elsa Sfeir, Chadia Haddad, Marwan Akel, Souheil Hallit, Sahar Obeid

**Affiliations:** 1 Faculty of Medicine and Medical Sciences, Holy Spirit University of Kaslik (USEK), Jounieh, Lebanon; 2 Tropical Neuroepidemiology, Institute of Epidemiology and Tropical Neurology, INSERM, Univ. Limoges, IRD, GEIST, U1094 Limoges, France; 3 Research Department, Psychiatric Hospital of the Cross, Jal Eddib, Lebanon; 4 INSPECT-LB (Institut National de Santé Publique, D’Épidémiologie Clinique Et de Toxicologie-Liban), Beirut, Lebanon; 5 School of Pharmacy, Lebanese International University, Beirut, Lebanon; 6 Faculty of Arts and Sciences, Holy Spirit University of Kaslik (USEK), Jounieh, Lebanon

**Keywords:** Sleep disturbance, Child, Diet, Nutrition, Depression, Anxiety

## Abstract

**Background:**

Sleep habits are an important component of a child’s health and it is affected by parent–child relationship. Also, child’s diet and nutrition appear to be an important factor affecting sleep health. Few studies have addressed the effect of parental emotional disturbance that can leave on children’s sleep. Therefore, the objective of our study was to assess the prevalence of sleep disorders in pre- and school-aged children and evaluate its relation with parental mental health and child’s nutrition and activity.

**Methods:**

A cross-sectional study, conducted between October 2020 and January 2021, which enrolled 402 Lebanese parents from all over Lebanon. The questionnaire was distributed online using the snowball technique. The Pediatric Sleep Questionnaire (PSQ) was used to assess pediatric sleep behaviors and the Family Nutrition and physical activity questionnaire was used to assess parental behaviors that might predispose children for obesity.

**Results:**

A total of 76 (19%) children had sleep disorders (PSQ scores of 8 or more). The multivariable analysis showed that higher paternal depression (Beta = 0.079, *p* = 0.010), maternal depression (Beta = 0.089, *p* = 0.001) and higher anxiety in the father (Beta = 0.064, *p* = 0.021) were significantly associated with higher PSQ scores (worse sleep) in the child. Higher Family Nutrition and Physical Activity Screening Tool scores in the child (Beta = -0.161, *p* < 0.001) was significantly associated with lower PSQ scores (better sleep).

**Conclusion:**

Paternal anxiety and depression, as well as maternal depression, were factors associated with children’s sleeping disorders. Future studies are needed to assess parental influence on child’s development.

## Background

Sleep disorders affect children and adolescents frequently. They are defined as disorders linked to the amount, duration or timing of sleep [1]. They are a dysregulation of the normal circadian rhythm, which is a biological rhythm that orchestrate every day’s sleep pattern [2]. Among the different types of sleep disorders, insomnia, obstructive sleep apnea, and circadian rhythm disorders are the most frequently studied [3]. Sleep habits are an important component of a child’s health. In fact, healthy development in the childhood period was linked to a good quality of sleep [4]. In addition, children cognitive functions, family wellbeing as well as academic performance were shown to be affected by childhood sleeping [5]. Despite this impact, many sleep problems pass unrecognized by healthcare providers [6]. If recognized, sleep disorders are lately diagnosed, and pass unappreciated until it interferes with child behavior mood or performance [7].

In fact, 25% to 50% of US children, and 40% of adolescents were described to face sleep problems in 2011 [8]. In 2016, a study conducted in the Middle East showed that 37% of parents reported that their child had a sleep problem [9]. A study conducted in Saudi Arabia in 2019 showed that 21% of children were at high risk of sleep disordered breathing [10]. Sleep disorders are highly prevalent in Lebanon with a percentage of 67.2% of Lebanese children [11]. In addition, the prevalence of severe sleep disorder among Lebanese children was found to be 5.6% in 2014 [11].

Sleep regulation was described to be affected by parent–child relationship. Furthermore, maternal depressive symptoms were cited to be a factor affecting sleep habits in children [12]. However, paternal influence of child sleep was less frequently studied [13].

Depression is a highly prevalent disorder that is characterized by excessive and persistent negative emotions or anhedonia, with negative cognition and or somatic dysfunction with impairment in daily functioning [14]. Depression is highly prevalent among mothers of child bearing age [15]. It was reported that one out of ten children are raised by depressed mothers [15]. Many reviews reported how depression in mothers negatively affects cognitive, interpersonal and emotional life of children, even the very young ones [5, 16]. It had been previously showed that children sleep disorders can predispose to parental depression [5]. From another perspective, a study conducted on infants showed that sleep disorders in infants can, by itself, be negatively influenced by maternal depression [17].

Rather than maternal influence on children sleep behaviors, studies are conducted to assess paternal influence given that paternal contribution to child functioning is on the rise. Paternal involvement was shown to lead to better sleep behavior in children. Few studies have addressed the effect paternal depression and anxiety can have on the child’s sleeping behaviors [4, 18].

Anxiety is another mental health problem that parents can face. It is a multisystem response to a perceived stress or danger. It can be a normal part of everyday life, but it can become pathological when it interferes with everyday activity. According to IDRAAC (Institute for Development, Research, Advocacy and Applied Care), 16.7% of Lebanese people will have an anxiety disorder in their lives [19]. Like depression, paternal anxiety can affect children’s sleep quality [20].

In addition to all the previously described predisposing factors for sleep disturbance in children, the child’s diet and nutrition appear to be an important factor affecting sleep health [3]. The mechanism by which nutrition affects the sleep pattern is very complex but can be resumed as follows: First, some food components can directly affect sleep by affecting sleep induction time. Other food metabolites can directly or indirectly contribute in sleep regulation. Finally, on the long term, nutritional elements can alter inflammatory biomarkers that were described to affect sleep patterns [21].

In the same perspective, children with increased physical activity appear to decrease adiposity, which can be related to better sleeping habits. However, the relation between physical activity in children and sleep disorders is not well established until now [22]. It was previously suggested that physical activity can be associated with better sleep habits [23], without knowing exactly the directionality of the relationship between sleep disorder and physical activity [24] (which factor affects the other).

Previous findings have examined the sleep habits and the prevalence of sleeping disorders in infants and children while few focused on the preschool- and school-aged children. However, sleep disorders in this age group can be clearly defined and can be more easily identified at a medical or behavioral level [25].

Depression and anxiety are highly prevalent in Lebanese population reaching 32.9% of its population [26]. This high rate of psychological disturbance in Lebanese people makes it important to highlight the impact they can leave on the wellbeing of Lebanese children.

Previous studies have discussed the effect of sleep disturbance in children, and some predisposing risk factors such as children obesity. Furthermore, it was previously described that parental anxiety and depression could moderate sleep disturbance in children. However, few studies assessed paternal and maternal emotional health as independent factors. To our knowledge, few studies addressed the effect parental emotional disturbance can leave on children’s sleep, and none in the Mediterranean region [27–29]. Given the very difficult socio-economic crisis Lebanese people are facing secondary to COVID-19 pandemic, on top of the increased prevalence of depression and anxiety after the blast that hit Beirut on August 4, 2020, it is highly important to highlight the effect of parental anxiety and depression on sleep pattern in children, given the social and emotional impact it can leave on children’s everyday life, as well on the learning disabilities it can leave [30]. It has previously been stated that culture can affect sleep pattern in children [31]. Bed sharing and co-sleeping were found to be more prevalent in countries with low socio-economic status and can affect sleep patterns in children [32]. Aside from bed sharing, many other factors were described to affect sleep patterns such as sleep parental behaviors, school schedule and local custom and traditions [31, 32]. Few studies assessed the effect Lebanese culture can have on Lebanese children’s sleep [33]. However, it has previously been described that Lebanese demographics and local customs such as late night evening meals can affect sleep pattern in Lebanese children [33].

To our knowledge, most research focus on insomnia in adults [33–37], with only few studies having addressed sleep disturbance in Lebanese pediatric population [11, 38, 39], and none assessed the effect parental psychological disturbance. The aim of our study was to assess the prevalence of sleep disorders in preschool- and school-aged children and evaluate its relation with parental mental health and child’s nutrition and activity.

## Methods

### Sampling and data collection

This was a cross-sectional study, conducted between October 2020 and January 2021, which enrolled 402 Lebanese parents from all over Lebanon. Due to the lockdown imposed by the Lebanese government during the coronavirus pandemic, the questionnaire was sent online (link:https://docs.google.com/forms/d/1rCESzq8E9JMPP7VxuaGu9sfcavjc1egHZCOBaYSmzFM/prefill). The link was distributed using the snowball technique. Parents had the choice to agree or to decline to participate in the study, with no monetary payment in exchange for their involvement. The questionnaire used was in Arabic, which is the Lebanese native language, and needed approximately 15 min to be completed. Families were asked to fill the questionnaire, with one part filled by the father, another one by the mother, and a part addressed to child behaviors filled by either parent. The Pediatric Sleep Questionnaire and Family Nutrition and physical activity questionnaire scales were translated from English to Arabic through an initial translation and a back-translation process. A certified translator translated the English version into Arabic; the latter version was translated back into English by a different translator. Discrepancies were resolved by consensus.

### Minimal sample size

According to the G-power software [40], and based on an effect size f2 = 2%, an alpha error of 5%, a power of 80%, and taking into consideration 20 factors to be entered in the multivariable analysis, the results showed that a minimal number of 395 was needed.

### Questionnaire

The first part was addressed to the mother; it assessed the sociodemographic characteristics (age, sex, educational level), and then assessed the daily activity of the mother, depression and anxiety. Fathers were asked to fill the second part, which was similar to the one addressed to the mother. The third part addressed the child’s gender and age. It then assessed the family nutrition and physical activity, and finally sleep pattern of the child. Parents having more than one child were asked to fill one form per child. Depression and anxiety among parents were assessed using Montgomery and Asberg Depression Rating Scale and the Lebanese Anxiety Scale. Sleep disturbance in children was assessed using pediatric sleep questionnaire.

### Montgomery and Asberg Depression Rating Scale (MADRS)

Montgomery Asberg Depression Rating Scale (MADRS), validated in Arabic [41], is a ten-item scale widely used for depression assessment. Questions are rated from a 0 to 6 (0 = no abnormality, 6 = severe), with higher scores indicating more depression.

### Lebanese Anxiety Scale (LAS-10)

Lebanese anxiety scale is composed of 10 items to assess the anxiety in Lebanese adults. Questions 1 to 7 are scored on a 5-point Likert scale from 0 (not present) to 4 (very severe), while items 8–10 are graded on a 4-point Likert scale. The higher the score, the higher the anxiety [42].

### Pediatric Sleep Questionnaire

It includes 28 items used to assess pediatric sleep behaviors. It was previously validated for the assessment of sleep-disordered breathing, snoring, sleepiness, and behavioral problems. Items are rates as yes, no, I do not know. Higher scores reflect more sleep problems. Eight “yes” answers and more suggest sleep disordered breathing, snoring, sleepiness and behavioral problems [43].

### Family Nutrition and physical activity questionnaire (FNPA)

It is a screening tool used to assess parental behaviors that might predispose children for obesity [44]. A total of 20 items, scored on a Likert scale from 1 to 4 (1 almost never, 2 sometimes, 3 usually, 4 almost always), compose this tool. Higher scores indicate better nutrition and physical activity in the child.

### Statistical analysis paragraph

The FACTOR software was used to conduct the factor analysis on the PSQ and FNPA, using the Pearson correlation matrix and using the parallel analysis as a procedure for determining the number of factors/components. The promax rotation was used to extract the items since the latter were highly correlated. The Kaiser–Meyer–Olkin (KMO) and the Bartlett’s test of sphericity p-value were calculated to ensure model’s adequacy.

Data analyses was done using the SPSS software v.25. The normality of distribution of the PSQ score was confirmed via a calculation of the skewness and kurtosis; values for asymmetry and kurtosis between -2 and + 2 are considered acceptable in order to prove normal univariate distribution [45]. These conditions consolidate the assumptions of normality in samples larger than 300 [46]. Accordingly, the Student t-test was used to compare two means (i.e., gender and marital status) while the ANOVA test was used to compare between three or more means (i.e., education level and monthly income). The Pearson correlation test was used to correlate two continuous variables. A stepwise linear regression was conducted taking the PSQ score as the dependent variable. All variables that showed an effect size or correlation coefficient > │0.24│ were entered in the multivariable analysis to have more parsimonious models [47]. Significance was verified at p < 0.05.

## Results

The sociodemographic characteristics of the participants are summarized in Table 1. The results showed that the mean age of the child was 8.02 ± 4.38 years and the mean ages of the mother and father were 36.5 ± 10.7 and 41.1 ± 11.6 years respectively. More than half of the child participants were females (57.1%) and the majority of the mothers and fathers had a university level of education. In addition, 76 (19.0%) children had sleep disturbances (PSQ scores of 8 or more).

**Table 1:** Sociodemographic characteristics of the study sample (*N* = 401)

	**Frequency**	**Percentage**
**Gender of the child**		
Female	208	57.1%
Male	156	42.9%
**Education level of the mother**		
Complementary or less	43	10.7%
Secondary	89	22.2%
University	269	67.1%
**Education level of the father**		
Complementary or less	61	15.2%
Secondary	72	18.0%
University	268	66.8%
	**Mean**	**SD**
**Age of the mother (in years)**	36.5	10.7
**Age of the father (in years)**	41.1	11.6
**Age of the child (in years)**	8.02	4.38

Some values might not round up to the total number of the sample because of missing values

### Description of the scales used in the study

Table 2 describes all the scales used in terms of mean, standard deviation, median, minimum and maximum.

**Table 2:** Description of the scales used in the study

	**Median**	**Mean**	**SD**	**Minimum**	**Maximum**
Anxiety in mother (LAS-10)	14.00	15.23	9.73	3	40
Depression in mother (MADRS)	8.00	10.04	10.40	0	44
Anxiety in father (LAS-10)	12.00	13.71	9.47	3	40
Depression in father (MADRS)	2.00	7.80	10.40	0	48
Family Nutrition and Physical Activity Screening Tool among child	54.00	55.44	7.45	36	72
Pediatric Sleep Questionnaire among child	2.00	3.80	4.77	0	21

### Factor analysis

A first factor analysis using the principal analysis component, was conducted on the total sample for the PSQ items. The KMO value (= 0.866) and the p-value of the test of sphericity (p < 0.001) confirmed the sample adequacy. Items converged over a two-factor solution, explaining a total variance of 51.07% (Cronbach’s alpha for the total scale = 0.929) (Table 3, Model 1).

**Table 3:** Factor analyses

**Model 1: Factor analysis of the Pediatric Sleep Questionnaire using the promax rotation (*N* = 401)**			
**SPQ item** While sleeping does your child…	**Sleepiness and attention / hyperactivity**	**Snoring and breathing problems**	
1. Snore more than half the time?		0.834	
2. Always snore?		0.969	
3. Snore loudly?		0.920	
4. Have “heavy” or loud breathing?		0.584	
5. Have trouble breathing or struggle to breathe?		0.602	
6. Seen your child stop breathing during the night?		0.513	
7. Tend to breathe through the mouth during the day?		0.474	
8. Have a dry mouth on waking up in the morning?	0.472		
9. Occasionally wet the bed?	0.582		
10. Wake up feeling un-refreshed in the morning?	0.870		
11. Have a problem with sleepiness during the day?	0.834		
12. Does your child have a teacher or other supervisor commented that your child appears sleepy during the day?	0.783		
13. Is it hard to wake your child up in the morning?	0.823		
14. Does your child wake up with headaches in the morning?	0.791		
15. Did your child stop growing at a normal rate at any time since birth?	0.500		
16. Is your child overweight?	0.427		
17. This child often does not seem to listen when spoken to directly	0.400		
18. This child often has difficulty organizing tasks Is easily distracted by extraneous stimuli Fidgets with hands or feet or squirms in seat	0.718		
19. This child often is easily distracted by extraneous stimuli	0.566		
20. This child often fidgets with hands or feet or squirms in seat	0.617		
21. This child often is “on the go” or often acts as if “driven by a motor”	0.556		
22. This child often interrupts or intrudes on others (e.g. butts into conversations or games)		0.365	
Percentage of variance explained	41.52	9.55	
Cronbach’s alpha	0.917	0.839	
**Model 2: Factor analysis of the Family Nutrition and Physical Activity questionnaire using the promax rotation (*N* = 401)**			
**FNPA items**	**Family eating pattern and quality**	**Family eating practice**	**Eating and physical activity**
1. How often does your child eat breakfast, either at home or at school?		0.779	
2. How often does your child eat at least one meal a day with at least one other family member?		0.801	
3. How often does your child eat while watching TV? [Includes meals or snacks]	0.511		
4 How often does your family eat “fast food?”	0.676		
5. How often does your family use packaged “ready-‐to-‐eat” foods? [Includes purchased frozen or on-‐the-‐shelf entrees, often designed to be microwaved]	0.790		
6. How often does your child eat fruits and vegetables at meals or snacks? [Not including juice]		0.734	
7 How often does your child drink soda pop or sweetened beverages? [Includes regular or diet soda pop, Kool-‐Aid, Sunny-‐D, Capri Sun, fruit or vegetable juice, caffeinated energy drinks (Monster/Red Bull), Powerade/Gatorade, etc.]	0.776		
8 How often does your child drink low-‐fat milk for meals or snacks? [Includes 1% or skim dairy, flavored, soy, almond, etc.]	0.525		
9 How often does your family monitor the amount of candy, chips, and cookies your child eats?		0.761	
10 How often does your family use candy, ice cream or other foods as a reward for good behavior?		0.430	
11 How often does your child have less than 2 h of “screen time” in a day? [Includes TV, computer, game system, or any mobile device with visual screens]		0.440	
12 How often does your family monitor the amount of “screen time” your child has?		0.497	
13 How often does your child engage in screen time in his/her bedroom?	0.688		
14 How often does your family provide opportunities for physical activity?			0.738
15 How often does your family encourage your child to be physically active?			0.725
16. How often does your child do physical activities with at least one other family member?			0.823
17 How often does your child do something physically active when he/she has free time?			0.796
18 How often does your child participate in organized sports or physical activities with a coach or leader?			0.695
19 How often does your child follow a regular routine for your child’s bedtime?		0.547	
20 How often does your child get enough sleep at night?		0.690	
Percentage of variance explained	32.84	15.12	8.42
Cronbach’s alpha	0.770	0.842	0.863

A second factor analysis using the principal analysis component, was conducted on the total sample for the FNPA items. The KMO value (= 0.883) and the p-value of the test of sphericity (p < 0.001) confirmed the sample adequacy. Items converged over a two-factor solution, explaining a total variance of 56.38% (Cronbach’s alpha for the total scale = 0.864) (Table 3, Model 2).

### Bivariate analysis

The results of the bivariate analysis, taking the pediatric sleep questionnaire as the dependent variable, showed that a lower mean PSQ score was found in parents having a university level of education compared to the other groups. In addition, a higher parental age, higher anxiety and higher depression were significantly associated with higher PSQ (worse sleep) in the child. Higher Family Nutrition and Physical Activity Screening Tool scores in the child were significantly associated with lower PSQ scores (better sleep) (Table 4).

**Table 4:** Bivariate analysis taking the Pediatric Sleep Questionnaire among child as the dependent variable

	**Pediatric Sleep Questionnaire**	**Effect size d **_**Cohen’s**_	***p*-value**
	**Mean ± SD**		
**Gender of the child**			
Male	3.80 ± 4.95	0.01	0.924
Female	3.85 ± 4.66		
**Education level of the mother**			
Complementary	8.00 ± 5.56	0.34	** < 0.001**
Secondary	3.84 ± 4.35		
University	3.17 ± 4.43		
**Education level of the father**			
Complementary	6.73 ± 4.74	0.30	** < 0.001**
Secondary	5.38 ± 5.40		
University	2.92 ± 4.33		
	**Correlation coefficient**		
**Age of the mother**	0.138		**0.008**
**Age of the father**	0.194		** < 0.001**
**Anxiety in mother**	0.398		** < 0.001**
**Depression in mother**	0.431		** < 0.001**
**Anxiety in father**	0.412		** < 0.001**
**Depression in father**	0.463		** < 0.001**
**Family Nutrition and Physical Activity Screening Tool among child**	-0.422		** < 0.001**

Numbers in bold indicate significant *p*-values

### Multivariable analysis

The results of a stepwise linear regression, taking the Pediatric Sleep Questionnaire as the dependent variable, showed that higher paternal depression (Beta = 0.099, *p* = 0.001), higher maternal depression (Beta = 0.064, *p* = 0.024) and higher anxiety in the mother (Beta = 0.059, p = 0.024) were significantly associated with higher PSQ scores (worse sleep) in the child. Higher Family Nutrition and Physical Activity Screening Tool scores in the child (Beta = -0.148, *p* < 0.001) were significantly associated with lower PSQ scores (better sleep) (Table 5).

**Table 5:** Multivariable analysis: Linear regression taking the Pediatric Sleep Questionnaire variable as the dependent variable

	**Unstandardized Beta**	**Standardized Beta**	***p*-value**	**95% Confidence Interval**	
				**Lower bound**	**Upper bound**
Depression in the father	0.099	0.214	0.001	0.043	0.154
Family Nutrition and Physical Activity Screening Tool in the child	-0.148	-0.231	< 0.001	-0.209	-0.086
Depression in the mother	0.064	0.140	0.024	0.008	0.120
University level of education in the father vs complementary or less*	-1.470	-0.139	0.002	-2.414	-0.525
Anxiety in the mother	0.059	0.120	0.024	0.008	0.109
Secondary level of education in the mother vs complementary or less*	-1.184	-0.095	0.030	-2.249	-0.118

Variables entered in the model: maternal level of education, paternal level of education, anxiety and depression in the mother and father and Family Nutrition and Physical Activity Screening Tool in the child

## Discussion

This study results demonstrated that paternal anxiety and depression as well as maternal depression, were factors associated with children’s sleeping disorders. In addition, our study highlighted the fact that higher family nutrition and physical activity was associated with less sleep disorders in children.

In this study, we found that 19% of children experienced sleep disorders. Those results were similar to those found in a study conducted in Saudi Arabia showing that 21% of children were at risk of sleep-disordered breathing [10]. This prevalence is lower than the overall prevalence estimated at 67.2% by a previous study conducted in Lebanon [11]. This can be explained by the fact that in our study, we assessed primary sleeping disorders related to breathing snoring, and behavioral problems among all sleeping disorders, given that those disorders put children at high risk of obstructive sleep apnea [43]. In addition, the scales used in both studies are different, possibly leading to a difference in prevalence of sleep disorders.

In our study, maternal anxiety and depression were associated with worse sleep in children. Those results are important, because they highlight the effect of maternal influence on children’s growth and wellbeing. Those results were similar to previous studies focusing on the effect of maternal depression on childhood behaviors and sleep disturbance [48], This can be explained by the fact that the mother is considered as the primary caregiver of the child [49]. Higher depressive symptoms in the mother can predispose to higher family interpersonal relationship insecurity and consequently higher emotional fragility in children. Consequently, children might feel unsecure, with higher level of vigilance about the future. This emotional insecurity can lead to a difficulty in falling and maintaining sleep [50–52]. In addition, maternal anxiety can influence on parenting skills of the mother, and can lead to negative parental cognition. Those negative cognitions can negatively affect maternal bedtime and nighttime behaviors in many event such as letting the child sleep late, or using an inconsistent bedtime routine [48].

Maternal depression has been extensively studied, and is described as a public health concern, given the high prevalence of depression among mothers especially during the postpartum period [53], and the negative impact maternal depression can leave on the emotional and cognitive development of the child [54, 55]. We showed in our study, that maternal depression was associated with sleep disturbance in children. Our results were similar to those in a previous study that showed that maternal depression can have a direct influence on the infant’s sleep pattern especially in the post-partum period [54]. In addition, a study assessing sleep disorder in children and maternal depression showed that maternal depression can be influenced by child sleeping problem [55]. In fact, children were shown to be highly aware of their mother’s emotions; this can lead to excess worrying at bedtime and can predispose to disturbance in sleeping patterns [12]. Furthermore, similarly to paternal depression, maternal depression can have a negative impact on the mother-father relationship, leading to higher emotional disturbance that can be a mediating factor for this association [56].

Finally, we found in our study that higher nutrition and physical activity scores in children were associated with better sleeping patterns [57]. In fact, this score was inversely correlated with child obesity [58]. Better nutrition habits, and healthier life style can lead to less obese children, and lower rates of sleep apnea and consequently better sleep pattern in children. It was previously described that children’s sleep apnea was highly associated with obesity [58]. In addition, higher physical activity rather than leading to better weight health in children, was described as a factor associated with better mental health and consequently better sleep habit [59, 60].

### Clinical implications

Sleep disorders in children were described to be highly prevalent among Lebanese children; if they pass unrecognized, they can negatively affect the child’s emotional wellbeing, school performance, and health. Given the high rates of depression and anxiety in the Lebanese population, our findings came to highlight the effect parental mental health can leave on the child’s wellbeing, particularly sleeping patterns, and consequently incite pediatricians to be more aware of sleep disturbance in Lebanese children and its predisposing factors. Furthermore, this study lightened up the need to implement workshops to raise awareness about sleep disorders in Lebanese children. In addition, those results might serve as a first step to encourage pediatricians to ask parents about their children’s sleep in order to discover any undiagnosed sleep disturbance and implement adequate strategies for a better quality of sleep in children.

### Limitations

Given our study is a cross sectional study, it can only assess factors correlated with sleep disturbance without being able to establish causality and directionality of relationship. In addition, a residual confounding bias is also possible since not all factors associated with sleep disturbance were taken into consideration in our study (such as children’s behaviors eating). Given that data is reported by parents, this study can be limited by response/information bias. Furthermore, given the wide range of children age assessed, results should be considered according to developmental differences, age-appropriate parental responsibilities and bedtime interaction patterns. Although the factor analyses results of the Arabic versions of the PSQ and FNPA scales were satisfactory, more psychometric properties are needed for both scales (convergent validity, divergent validity, etc.); therefore, our study results should be interpreted with caution. The sample was collected from all governorates, despite the use of the snowball technique (without randomization). However, weighting could not be applied to the analysis since we lack national numbers concerning the stratification by age group, gender and governorate in this age category.

## Conclusion

We found in our study that 19% of a sample Lebanese children had sleep disordered breathing. Maternal depression, paternal anxiety and depression were showed to be factors associated with more sleep disorders in children. In addition, more physical activity and good nutrition habits were associated with better sleeping patterns. This study highlights the effect of paternal emotional wellbeing on children’s health, particularly sleeping patterns, and opens up for future research assessing parental influence on sleep patterns.

## Data Availability

All data generated or analyzed during this study are included in this published article.

## Abbreviations

PSQ: Pediatric Sleep Questionnaire
IDRAAC: Institute for Development, Research, Advocacy and Applied Care
MADRS: Montgomery and Asberg Depression Rating Scale
LAS: Lebanese Anxiety Scale
